# A novel and effective method to generate human porcine-specific regulatory T cells with high expression of IL-10, TGF-β1 and IL-35

**DOI:** 10.1038/s41598-017-04322-3

**Published:** 2017-06-21

**Authors:** Mingqian Li, Judith Eckl, Christiane Geiger, Dolores J. Schendel, Heike Pohla

**Affiliations:** 1 Laboratory of Tumor Immunology, LIFE Center, Ludwig-Maximilians-Universität, Munich, Germany; 2 Department of Urology, University Hospital, Ludwig-Maximilians-Universität, Munich, Germany; 3 Institute of Molecular Immunology, HelmholtzZentrum München, German Research Center for Environmental Health, and Clinical Cooperation Group “Immune Monitoring”, Munich, Germany; 4 Medigene Immunotherapies GmbH, Planegg, Martinsried Germany

## Abstract

Organ transplantation remains the most effective treatment for patients with late stage organ failure. Transgenic pigs provide an alternative organ donor source to the limited availability of human organs. However, cellular rejection still remains to be the obstacle for xenotransplantation. Superior to other methods, antigen-specific regulatory T cells (Treg) alleviate cellular rejection with fewer side effects. Here we demonstrate the use of a fast method to provide tolerogenic dendritic cells (tolDC) that can be used to generate effective porcine-specific Treg cells (PSTreg). TolDC were produced within three days from human monocytes in medium supplemented with anti-inflammatory cytokines. Treg were generated from naïve CD4^+^ T cells and induced to become PSTreg by cocultivation with porcine-antigen-loaded tolDC. Results showed that PSTreg exhibited the expected phenotype, CD4^+^CD25^+^CD127^low/−^ Foxp3^+^, and a more activated phenotype. The specificity of PSTreg was demonstrated by suppression of effector T cell (Teff) activation markers of different stages and inhibition of Teff cell proliferation. TolDC and PSTreg exhibited high expression of IL-10 and TGF-β1 at both protein and RNA levels, and PSTreg also highly expressed IL-35 at RNA levels. Upon restimulation, PSTreg retained the activated phenotype and specificity. Taken together, the newly developed procedure allows efficient generation of highly suppressive PSTreg.

## Introduction

The shortage of human organs and cells remains a major obstacle for human organ transplantation. Genetically-engineered porcine organs have provided an alternative organ resource^1, 2^. Although tissue reprogramming alleviates organ hyper-rejection^3, 4^, the subsequent cellular rejection still needs to be overcome^5^. Several groups reported that immunosuppression therapy prolongs xeno-organ survival^6, 7^, but high dose administration of immune suppressive drugs is associated with severe side effects. Therefore a better tolerated and effective means to alleviate xeno-reactions is urgently needed and is the key step to be resolved for clinical xeno-transplantation applications in the future.

Dendritic cells (DC), first defined by Ralph M. Steinman and Zanvil A. Cohn in 1973^8^, are the most potent antigen-presenting cells. Tolerogenic DC (tolDC) maintain a semi-mature status and mediate tolerance *in vivo*. Accumulated evidence indicates that adoptive transfer of tolDC reverses graft versus host disease (GVHD)^9, 10^. Further studies revealed that tolDC exhibit their tolerogenic function via Treg^11^, the generation of which relies on DC–T cell contact *in vivo* ^12^.

In humans, Treg represent cells that maintain homeostasis and are defined as a subset of CD4^+^ T cells that express high levels of CD25 (IL-2 receptor α-chain) and forkhead box P3 (FoxP3) and a low level of CD127. Treatments with Treg proved effective in alleviating allergy, rebuilding immune-balance for type 1 diabetes and reducing GVHD^13–15^. However, *ex vivo* expansion of natural Treg (nTreg) is expensive and laborious^16^, while, in contrast, antigen-specific induced Treg exhibit highly potent suppression at low cell numbers^17^. Therefore, development of fast and effective methods to generate xeno-specific Treg with highly suppressive properties would fulfill an urgent medical need. Human Treg can reverse a xeno-reaction which was first reported by Porter and Bloom in 2005^18^. In 2008, Wu *et al*. reported that human porcine specific Treg were generated from nTreg using irradiated porcine peripheral blood mononuclear cells as stimulator cells^19^. Adoptive transfer of porcine specific Treg in humanized mice can inhibit xenograft rejection^20^.

IL-12 family members are heterodimeric cytokines including IL-12p70, IL-23, IL-27 and IL-35. IL-12p70 is composed of IL-12p35 and IL-12p40 subunits and is produced by DC. It mediates IFN-γ, TNF-α and IL-2 production and induces Th1 cell differentiation. IL-35 is a novel IL-12 family cytokine which received much attention because of its function in immune tolerance. IL-35 consists of EBV-induced gene 3 (EBI3) and IL-12p35 subunits. IL-35 was found to be constitutively expressed at high levels in mouse Foxp3^+^ Treg^21^, and several groups showed that IL-35 promotes regulatory B cell and Treg proliferation and mediates suppressive functions^21–23^. In contrast, in humans, IL-35 is only expressed in activated Treg^24^. IL-27 shares the β-chain (EBI3) with IL-35; the α-chain of IL-27 is IL-27p28 (IL-27A). IL-27 is an immune regulatory cytokine that induces Th17 cells to produce IL-10^25, 26^, but it also exerts anti-tumor effects by enhancing survival of tumor-antigen-specific CD8^+^ T cells^27^ and suppressing Treg by blocking the expression of Foxp3 via STAT1 and STAT3 activation^28, 29^.

In this paper, we present a new method for generation of highly suppressive xeno-reactive Treg that secrete high amounts of IL-10, IL-35 and TGF-β1 through cocultivation of naïve CD4^+^ T cells with porcine-antigen-loaded tolDC. The highly suppressive properties and specificity of the xeno-reactive Treg were demonstrated using different functional assays and their cytokine secretion pattern of IL-10, TGF-β1 and IL-12 family members was well demonstrated.

## Results

### Tolerogenic DC express B7-H1, B7-DC, IL-10 and TGF-β1

To generate tolDC and immunogenic DC (C5-DC)^30^, fresh CD14^+^ monocytes were isolated from healthy donor peripheral blood mononuclear cells (PBMC) (Supporting Information Fig. S1a) and anti-inflammatory or inflammatory cytokines were added to induce tolerogenic or immunogenic phenotypes, respectively.

**Figure 1:**
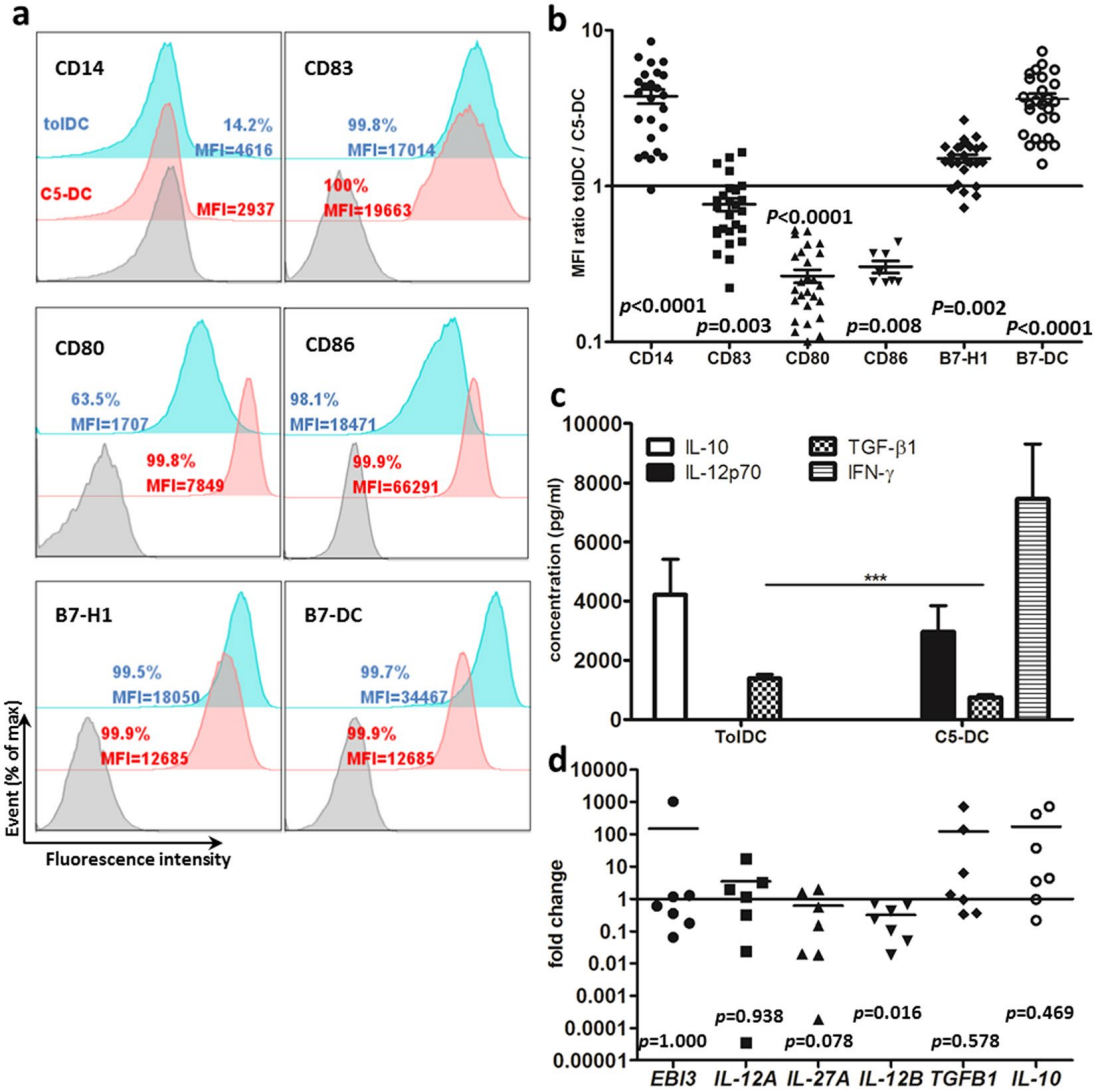
DC generation from monocytes with anti-inflammatory cytokines exhibited tolerogenic phenotype and produced anti-inflammatory cytokines. TolDC and C5-DC were harvested after maturation and characterized by flow cytometry, anti-CD14, anti-CD83, anti-CD80, anti-CD86 anti-B7-H1 and anti-B7-DC antibodies were used. (**a**) Histogram, (**b**) dot chart of MFI ratio tolDC to C5-DC. Data represent 26 independent experiments from 9 donors (CD86: n = 8). Wilcoxon signed-rank test was used to determine p values. (**c**) IL-10, IL-12p70, TGF-β1 and IFN-γ quantification in DC supernatants using BD CBA Flex Set and ELISA. These data represent 10–19 independent experiments from 9 donors. Culture medium plus maturation cocktail without cells were used as control. Turkey test was used to determine the statistical significance. ***p < 0.001. RNA was isolated from tolDC and C5-DC, and quantified by RT-PCR, (**d**) Relative RNA level of *IL-35* and related genes, *IL-10*, *TGFB1* in tolDC, C5-DC as control. *ACTB*, *G6PDH or CYPB* mRNA were determined from the same sample and used as endogenous reference genes. These data represent 7 independent experiments from 6 donors. Wilcoxon signed-rank test was used to determine p values. Error bars: s.e.m.

TolDC retained some CD14 expression compared with C5-DC, which totally lost CD14 expression after maturation. On the other hand, tolDC expressed significantly lower levels of CD83 compared with C5-DC. These results support the conclusion that tolDC are semi-mature cells (Fig. 1a,b)^31^. CD80 and CD86, as costimulation molecules of the B7 family, were expressed at significantly lower levels on tolDC compared to C5-DC, showing 4-fold and 3.5-fold decrease compared to C5-DC, respectively. In contrast, the negative costimulators B7-H1 and B7-DC were significant highly expressed on tolDC compared to C5-DC. We detected 1.5- and 4-fold higher levels of expression of B7-H1 and B7-DC, respectively, on tolDC in comparison to C5-DC (Fig. 1a,b).

To evaluate tolDC function, IL-10, TGF-β1, IL-12p70, IFN-γ, IL-27 and IL-35 cytokine production was quantified with BD™ CBA Flex Set system, ELISA (enzyme-linked immunosorbent assay) and quantitative RT-PCR. As expected, at the protein level, tolDC produced a significantly higher amount of IL-10 and TGF-β1, and no IL-12p70 and IFN-γ when compared with C5-DC, while C5-DC produced greater amounts of IL-12p70 and IFN-γ, less TGF-β1, and no IL-10 (Fig. 1c). Consistently, *IL-10* mRNA levels were also upregulated in most tolDC of different donors, although the data did not meet statistical significance. Half of the samples showed elevated *TGFB1* RNA levels in tolDC compared to C5-DC (Fig. 1d). Generally, *EBI3* and *IL-12A* mRNA expression showed no significant difference in tolDC compared with C5-DC. *IL-27A* expression was downregulated in tolDC compared with C5-DC, however with a p-value of only 0.078. *IL-12B* was significantly downregulated in tolDC, which was consistent with the protein level results showing that C5-DC expressed high amounts of IL-12p70 while tolDC produced no IL-12p70 (Fig. 1d).

In summary, our tolDC express high levels of IL-10, TGF-β1, and no IL-12p70 and IL-27.

### DC express porcine antigen following electroporation with porcine *in vitro* transcribed RNA (ivtRNA)

**Figure 2:**
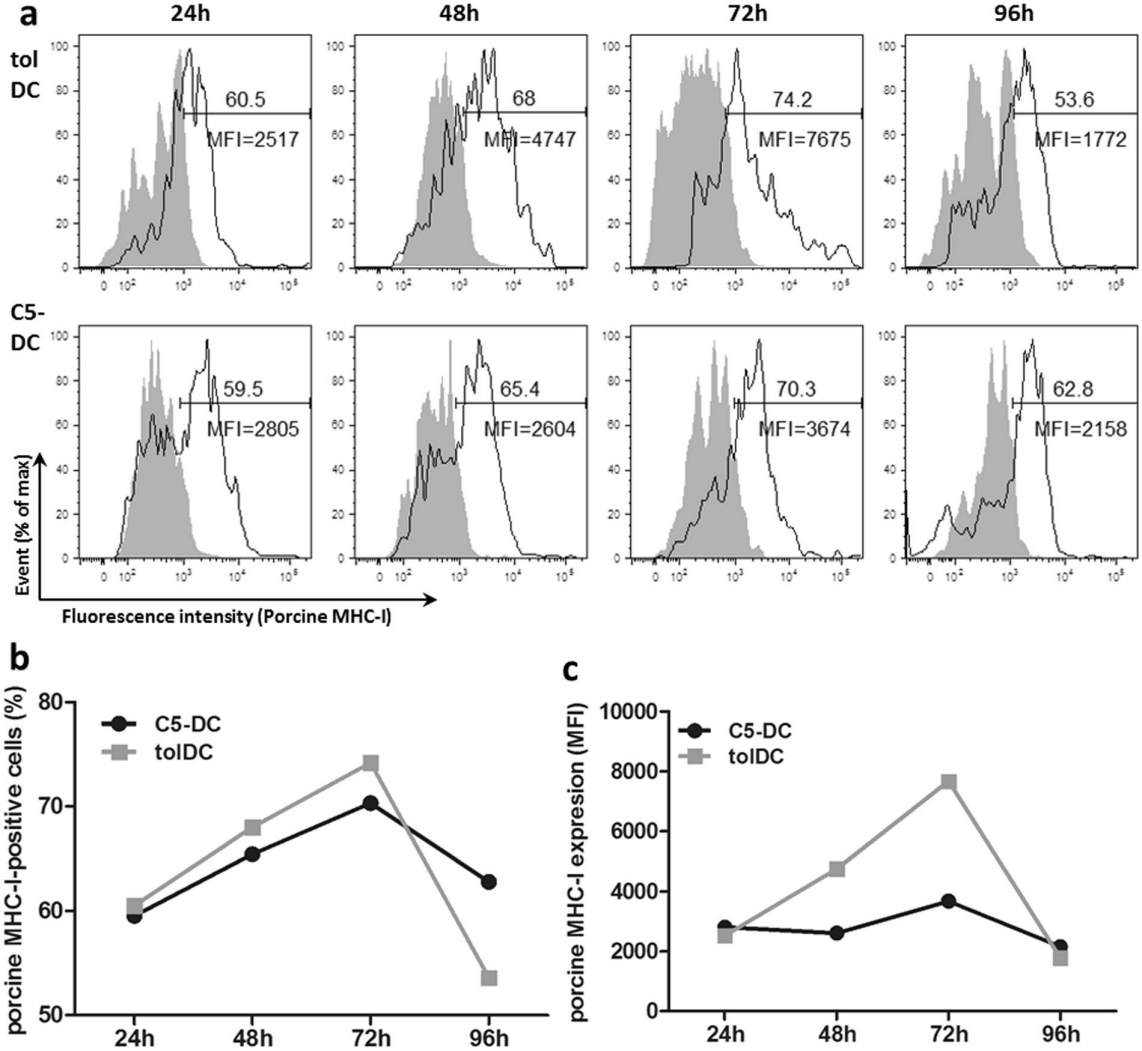
TolDC and C5-DC successfully express porcine antigen following electroporation of porcine-specific ivtRNA. MHC-I expression was used as surrogate marker for porcine antigen expression. (**a**) Flow cytometry analysis at different time points after electroporation using a porcine MHC-I-specific monoclonal antibody. Mock-electroporated tolDC and C5-DC were used as controls (grey peaks). (**b**) in % of cells, (**c**) as MFI.

Electroporation of xenogeneic mRNA into DC is a rapid way to induce foreign antigen production in DC. However, unlike transfection of a specific mRNA, the efficiency of transfection of total cellular mRNA is difficult to assess in host cells. In these studies we used detection of porcine MHC-I molecules as a surrogate marker of porcine specific (PS) antigen expression in DC. The quality of porcine PBMC RNA, cDNA and ivtRNA was controlled with capillary or agarose gel electrophoresis (Supporting Information Fig. S2). Detection of porcine MHC-I expression by flow cytometry indicated that PS ivtRNA was successfully transfected and expressed in tolDC and C5-DC. As expected, PS antigen expression from ivtRNA was transient: MHC-I antigen became detectable on both tolDC and C5-DC after 24 h and peaked at 72 h (Fig. 2a–c). The viability was shown in the Supporting Information (Fig. S3).

### PSTreg and Teff (PSTeff) can be generated with tolDC and C5-DC, respectively

PSTreg were induced from CD4^+^ naïve T cells, the purity of which was confirmed by flow cytometry (Supporting Information Fig. S1b), through coculture with PS-antigen-loaded tolDC supplemented with high concentration IL-2 and addition of rapamycin. In parallel, PSTeff were generated from CD4^+^ naïve T cells through coculture with porcine-antigen-loaded C5-DC, supplemented with a lower concentration of IL-2 and without rapamycin.

**Figure 3:**
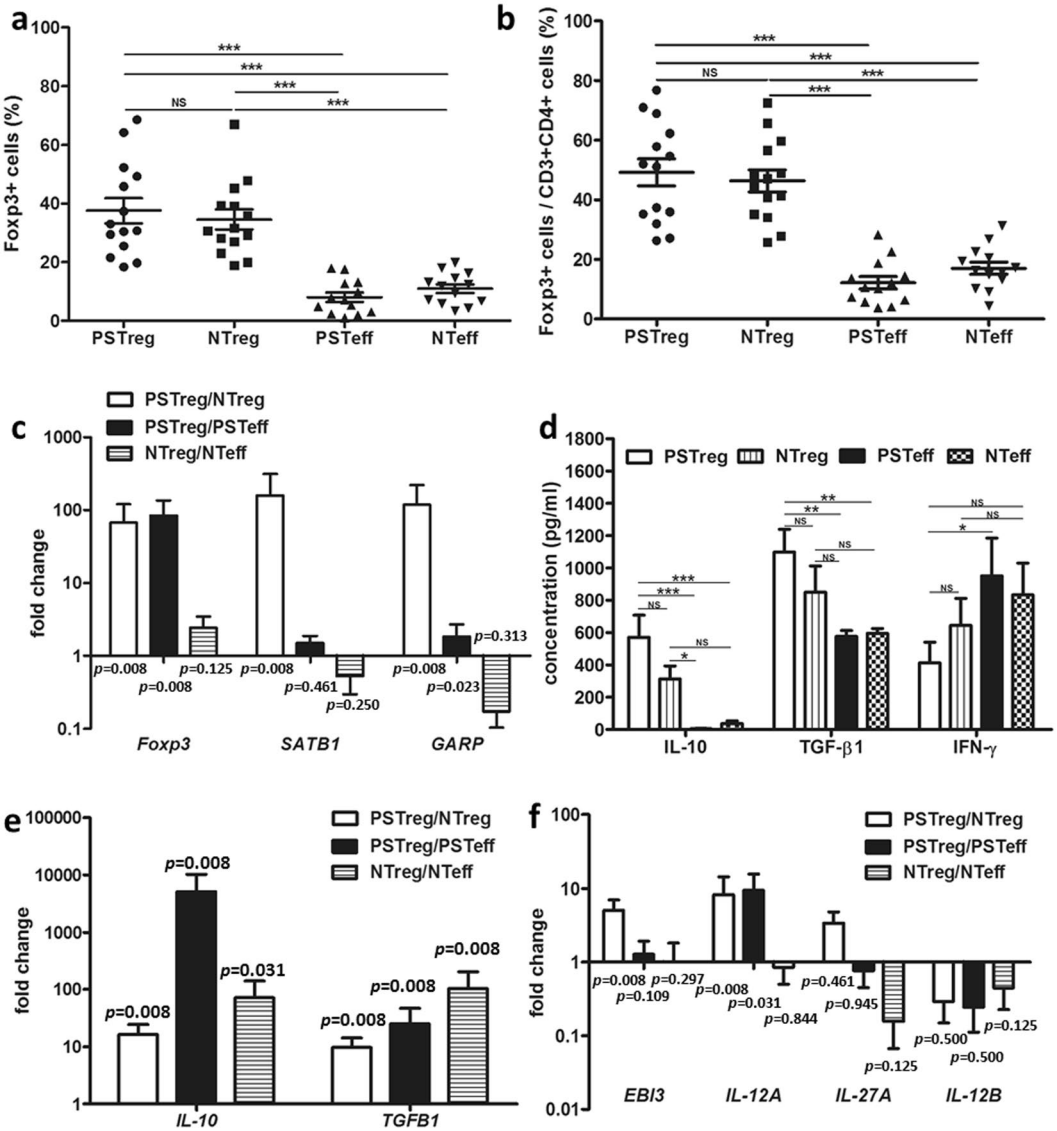
High IL-10, TGF-β1 and IL-35 secreting PS Treg generated with porcine antigen loaded DC. CD4^+^ T cells were isolated and cocultured with PS antigen-expressing DC for 10 days in the presence of IL-2 w/o rapamycin. (**a**,**b**) Foxp3^+^ cells in PSTreg and NTreg in the alive cell population and in the CD3^+^CD4^+^ cell population after 10 day coculture. For comparison PSTeff and NTeff were analyzed. Data represent 14 independent experiments with cells from 8 donors. (**c**) Relative quantification of *Foxp3*, *SATB1* and *GARP* mRNA of PSTreg and NTreg by RT-PCR. Data represent 8 independent experiments from 6 donors. (**d**) Supernatants of coculture were harvested for analyzing IL-10, TGF-β1, and IFN-γ. Data represent 20 experiments from 7 donors. (**e**) Relative expression of *IL-10*- and *TGFB1*-mRNA expression. Data represent 8 experiments from 6 donors. (**f**) Relative expression of IL-35-, IL-27-, and IL-12p70 related mRNA. Data represents 8 independent experiments from 6 donors. (**c**,**e**,**f**) represent NTreg and PSTeff as control for PSTreg and NTeff as control for NTreg. *ACTB*, *G6PDH or CYPB* mRNA were determined from the same sample and used as endogenous reference genes. ***p < 0.001, **p < 0.01, *p < 0.05. NS, non-significant, p > 0.05. ANOVA with Bonferroni’s Multiple Comparison Test or turkey test was used to determine the statistical significance of Treg percentage (**a**,**b**), and cytokine production on protein level (**d**). Wilcoxon signed-rank test was used to determine statistical significance of mRNA fold change (**c**,**e**,**f**). Error bars: s.e.m.

PSTreg and non-specific Treg (NTreg) exhibited the conventional human CD4^+^CD25^+^CD127^low/−^ Foxp3^+^ phenotype (Supporting Information Fig. S4a). There was no significant difference in the fraction of Foxp3-positive PSTreg and NTreg in both the CD3^+^CD4^+^ cells and the total cell populations (Fig. 3a,b, Table 1), PSTreg expressed significantly more *Foxp3* mRNA than NTreg (Fig. 3c). However, NTreg inhibited *SATB1* mRNA expression, which at low expression is considered as a new marker of Treg, compared with PSTreg and non-specific Teff (NTeff) (Fig. 3c). GARP is known as an activation marker of Treg: PSTreg expressed significantly more *GARP* mRNA than NTreg (Fig. 3c). Furthermore, PSTreg exhibited a memory phenotype compared with NTreg, showing CD45RA^−^Foxp3^+^, and expressed significantly lower levels of CCR7 (Supporting Information Fig. S4b,c). Moreover, CCR4 was significantly upregulated on PSTreg compared with NTreg (Supporting Information Fig. S4c). Similar to PSTreg, PSTeff also showed a more prominent memory phenotype (CD45RA^−^CCR7^low^) than NTeff (Supporting Information Fig. S4d,e).

**Table 1:** Frequency of CD4^+^CD25^+^CD127^low/−^ Foxp3^+^ cells in PSTreg and NTreg and PSTeff and NTeff populations.

	PSTreg	NTreg	PSTeff	NTeff
Treg in CD3^+^CD4^+^ cell fraction (%)	49.1 ± 4.5	46.3 ± 3.7	12.1 ± 2.1	17.0 ± 2.0
Treg in live cell fraction (%)	37.5 ± 4.3	34.5 ± 3.4	8.1 ± 1.6	11.0 ± 1.5

To characterize cytokine production of PSTreg, culture supernatants of cocultures were harvested after coculture for 10 days. PSTreg expressed significantly higher concentrations of IL-10 and TGF-β1 compared with both NTeff and PSTeff (Fig. 3d). Although not statistically significant they also showed higher concentrations than found for NTreg. PSTeff expressed the highest levels of IFN-γ, which was significantly higher than that found for PSTreg (Fig. 3d). IL-17A, an indicator of plasticity and instability of Treg, was undetectable in PSTreg and NTreg (data not shown). IL-12p70 was also not expressed in PSTreg and NTreg (data not shown). Expression levels of *TGFB1* and *IL-10* mRNA in PSTreg were significantly higher than in NTreg and PSTeff, which showed 7.5- and 23.6- fold and 16.4- and 5071-fold higher than the expression levels of NTreg and PSTeff, respectively. NTreg also upregulated significantly more *TGFB1* and *IL-10* mRNA than NTeff. These results were consistent with those obtained at the protein level (Fig. 3d,e).

PSTreg expressed significantly more *EBI3* and *IL-12A* mRNA compared to NTreg, and significantly more *IL-12A* mRNA compared to PSTeff (Fig. 3f). This indicates that PSTreg produce more IL-35 than NTreg. PSTreg did not express significantly higher amounts of *IL-27A* mRNA in comparison to NTreg and PSTeff. Both PSTeff and NTeff expressed 47.1- and 843-fold more *IL-12B* mRNA than PSTreg and NTreg, however, the data did not meet statistical significant because only 4 samples out of 8 contained detectable *IL-12B* RNA (Fig. 3f).

To test the stability of PSTreg, the purified PSTreg and NTreg cells were restimulated with PS antigen loaded or mock loaded tolDC or C5-DC. Upon restimulation, PSTreg maintained Foxp3 expression (Supporting Information Fig. S5) and kept the more prominent memory phenotype than NTreg (Supporting Information Fig. S6a,b): CD45RA^−^CCR7^low^Foxp3^+^, and expressed significantly higher levels of CCR4 than NTreg (Supporting Information Fig. S6b). NTreg obtained also CD45RA^−^Foxp3^+^ phenotype upon restimulation, but at a significant lower level than PSTreg. PSTeff also maintained the more prominent memory phenotype than NTeff: CD45RA^−^CCR7^low^ (Supporting Information Fig. S6c,d).

By depletion of nTreg, residual CD4^+^ T cells were used as precursor cells to generate PSTreg. PSTreg can also be generated from these cells, and no significant difference in suppressive function was observed (Supporting Information Fig. S7a–d).

These results confirmed that CD4^+^CD25^+^CD127^−/low^Foxp3^+^CD45RA^−^CCR7^low^CCR4^high^GARP^high^ PSTreg with high IL-10, TGF-β1 and IL-35 expression could be generated with our method.

### PSTreg demonstrate specific immunosuppressive activity

**Figure 4:**
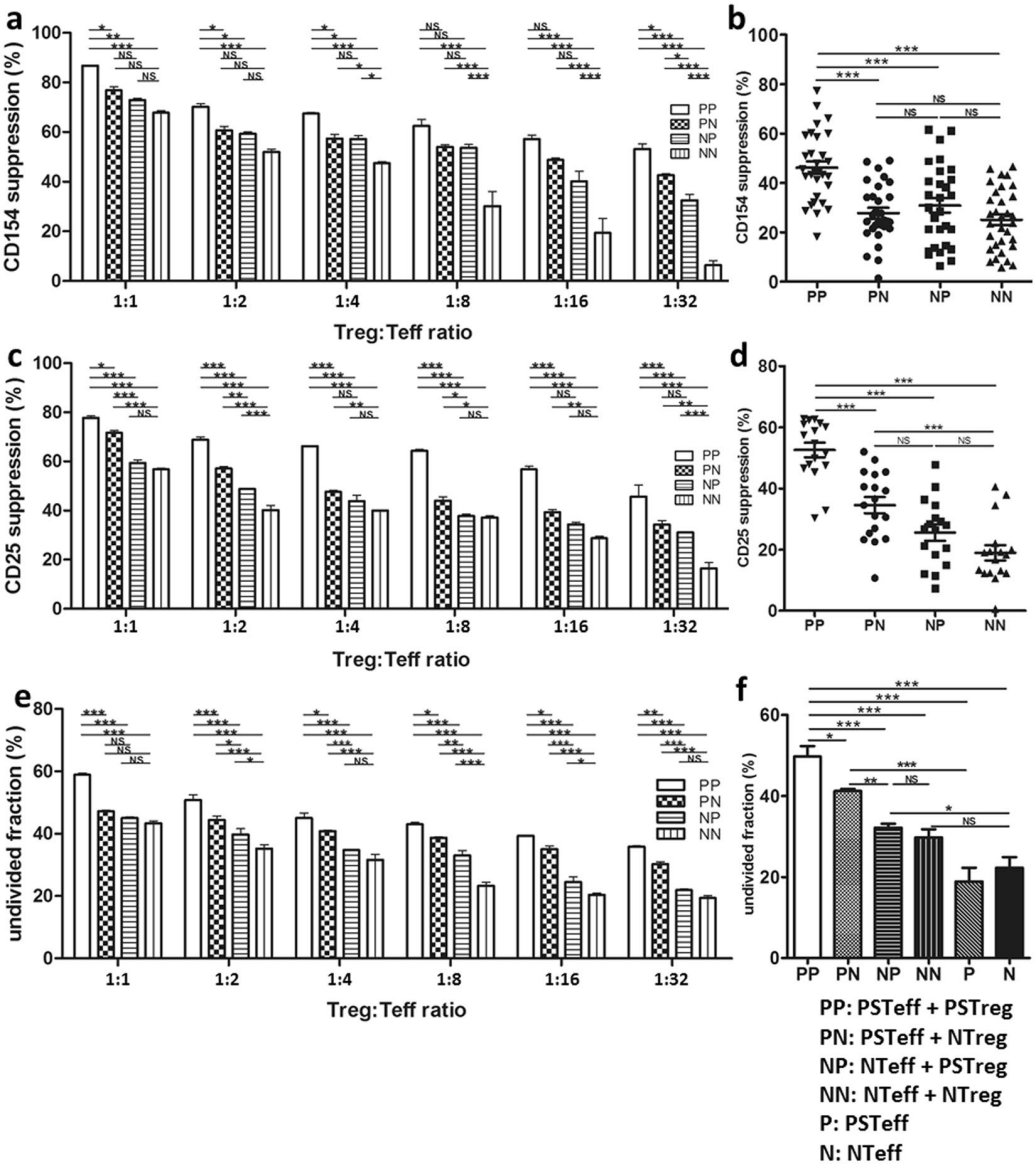
PSTreg show specific immunosuppressive activity. Three different assays were used to test PSTreg suppressive function towards PSTeff. PS/NTreg and PS/NTeff were seeded in 96 U-well plates with CD3/CD28 beads at different ratios, NTeff and PSTeff were set up as controls. Anti-CD154 and anti-CD25 were used to determine the Teff activation suppression, and CellTrace™ CFSE Cell Proliferation Kit was used to determine the inhibition of Teff proliferation. (**a**) Suppression of CD154 after 7 h at different ratios. (**c**) Suppression of CD25 after 96 h. (**e**) Inhibition of Teff proliferation at different ratios. (n = 3). (**b**) Suppression of CD154 at ratio 1:1, (**d**) shows suppression of CD25 at ratio 1:1, Data represents 15 independent experiments from 8 donors and 10 independent experiments from 6 donors, respectively. (**f**) Inhibition of Teff proliferation at ratio 1:1, (**e**) Data represents 8 independent experiments from 5 donors. ***p < 0.001, **p < 0.01, *p < 0.05. NS, not significant (p > 0.05). ANOVA with Bonferroni’s Multiple Comparison Test was used to determine the statistical significance. Error bars: s.e.m.

CD154 is an early activation marker of Teff, which is expressed in the first few hours after Teff activation. After coculture of porcine-specific and non-specific Treg and Teff cells for 7 h, the cells were harvested and tested for CD154 expression in the PSTeff and NTeff cells by flow cytometry (Fig. 4a,b, Supporting Information Fig. S8a). Expression of CD154 of Teff was significantly suppressed in the PSTeff/PSTreg coculture group (PP) compared with the other groups: CD154 expression in PSTeff was suppressed by nearly 50% in the PP group at ratio 1:1 (Fig. 4b). PSTreg specific suppressive function towards PSTeff was observed in different ratios, and still remained at a ratio of 1:32 (Fig. 4a). However, CD154 suppression of PN groups and NP groups showed no significant difference at the different ratios, except ratio 1:32. In comparison with nTreg, PSTreg also exhibited a significant higher suppressive function towards PSTeff in the CD154 expression assay (Supporting Information Fig. S8d).

After 4 days of coculture, CD25, an intermediate activation marker of Teff, was measured to evaluate longer term regulatory function of PSTreg. As expected, the expression of CD25 was also suppressed significantly in the PP group compared to the other groups in different ratios, and PSTreg specific suppression was also retained at a ratio of 1:32 (Fig. 4c,d, Supporting Information Fig. S8b). However, PSTreg showed no significant suppressive function towards NTeff in the ratios 1:1, 1:4, 1:8, 1:16. PSTreg also demonstrated a significant higher suppressive function towards PSTeff in the CD25 expression assay compared to nTreg (Supporting Information Fig. S8e).

Likewise, in the T cell proliferation assay (Fig. 4e,f, Supporting Information Fig. S8c), the undivided population of Teff in the PP group was significantly higher than in the other groups at different ratios, which indicated that the proliferation of PSTeff was inhibited by PSTreg. As demonstrated above, PSTeff proliferation was significantly inhibited by PSTreg than by nTreg (Supporting Information Fig. S8f).

Restimulated PSTreg kept their specific suppressive function towards PSTeff with respect to the activation markers CD154 and CD25 (Supporting Information Fig. S6e,f).

These experiments revealed the high specificity of PSTreg towards PSTeff.

### PSTreg express high amounts of IL-10, TGF-β1, and IL-35 after interaction with PSTeff

To further evaluate PSTreg functionality, cell supernatants were harvested after the functional assay to measure cytokine expression.

**Figure 5:**
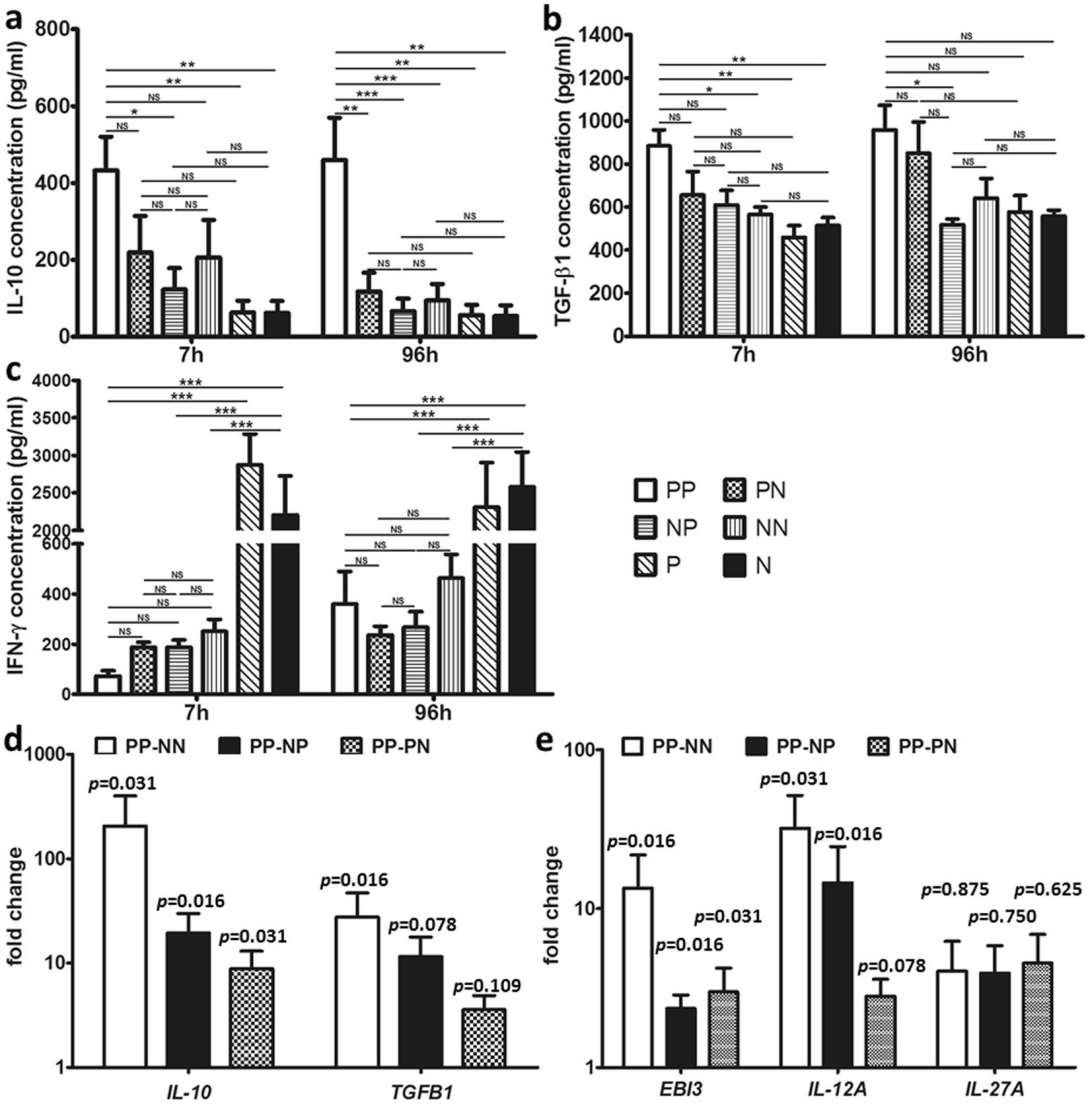
PSTreg express high amounts of IL-10 and TGF-β1. Supernatants from Treg and Teff cocultures were harvested after 7 h and 96 h. IL-10 (**a**), TGF-β1 (**b**) and IFN-γ (**c**) were quantified with BD™ CBA Flex Set analysis and ELISA. Data represents 4-14 independent experiments from 4–8 donors. ***p < 0.001, **p < 0.01, *p < 0.05, NS, non-significant, p > 0.05. ANOVA with Bonferroni’s Multiple Comparison Test was used to determine the statistical significance. The cells of the functional assay were also harvested after coculture for 96 h, and RNA was isolated and the indicated mRNA was quantified by RT-PCR. Relative expression of *IL-10*, *TGFB1* (**d**) and *IL-35* related mRNA (**e**) in PP to other groups was shown, PN, NP, NN as control. Data represents 7 independent experiments from 6 donors. *ACTB*, *G6PDH* or *CYPB* mRNA were determined from the same sample and used as endogenous reference genes. Wilcoxon signed-rank test was used to determine the p values.

As shown in Fig. 5a, high IL-10 levels were observed already after 7 h coculture of PSTreg with PSTeff. After coculture for 96 h, the amount of IL-10 in the supernatant increased slightly. In the PN, NP and NN coculture groups much lower levels of IL-10 were found.

In the first 7 h, TGF-β1 secretion in the PP group was significantly higher than in the NN coculture group, and in P and N cells. At the 96 h time point, almost all groups produced increased amounts of TGF-β1 except the NP group. Although the PP group maintained the highest production of TGF-β1, this was not statistically significant (Fig. 5b).

As expected, P and N cells secreted high amounts of IFN-γ after stimulation with CD3/CD28 beads (Fig. 5c). The production of IFN-γ was significantly inhibited in the PSTreg NTreg coculture group. In the first 7 h, PSTreg remarkably inhibited IFN-γ production of PSTeff, although not of statistical significance, while after 96 h incubation, all Treg with Teff coculture groups accumulated IFN-γ. IL-17A and IL-12p70 secretion was also measured in all groups. IL-12p70 production was only observed in a few samples of P and N cells (data not shown). All coculture groups failed to secrete IL-17A and IL-12p70 after stimulation with CD3/CD28 beads (data not shown).

As expected, consistent with protein data, *IL-10* mRNA expression of the PP group was significantly higher compared to other groups (Fig. 5d). Relative expression of *TGFB1* mRNA in the PP group was significantly higher upregulated compared to the NN group, however, the relative high expression compared to PN and NP groups did not meet statistical significance. The expression of both *EBI3* and *IL-12A* mRNAs, encoding the two components of IL-35, was significantly upregulated in the PP group compared to the other groups (except the relative expression of *IL-12A* compared to PN meets no statistical significance) (Fig. 5e). *IL-12A* mRNA was upregulated remarkably compared to *EBI3* mRNA. In contrast, *IL-27A* mRNA expression was not significantly upregulated in the PP group compared to the other groups, and in only 4 of the 7 Treg/Teff coculture samples *IL-27A* was expressed (Fig. 5e). We also measured *IL-12B* mRNA expression, which was not detected in the Treg and Teff groups (data not shown).

These results indicate that all three anti-inflammatory cytokines were upregulated at the mRNA level.

## Discussion

Several methods have been reported to generate tolDC and most require 6 or more days to obtain these cells. In this paper, we investigated a fast method that needs only 3 days to acquire functional tolDC. TolDC generated with this fast protocol retained a semi-mature state with preservation of CD14 expression and low level expression of CD83, CD80 and CD86. Elsewhere, high expression of B7-H1 and B7-DC was reported to be characteristic for tolDC^32^. B7-H1 and B7-DC, also termed PD-L1 and PD-L2 respectively, are ligands for PD-1^33^. PD-1 signal induces T cell inactivation via inhibition of TCR ligation by targeting PI3K/Akt and Ras/MEK/Erk pathway, and inhibits T cell proliferation by inhibiting cell cycle progression by Cdk2 regulation, and induce iTreg production via TGF-β independent Smad3 regulation^34^. Our tolDC expressed significantly high levels of B7-DC and B7-H1, but in few samples the tolDC showed downregulation of B7-H1, indicating some differences to the published reports of other tolerogenic DC. B7-H1 was found to be upregulated by IFN-γ^35, 36^, which may explain its higher expression in some samples of C5-DC that were found to produce IFN-γ. *In vivo*, Treg are generated by DC that provide few or no inflammatory cytokines and costimulatory signals, in the presence of low antigen levels^31^. Our tolDC shared these characteristics and were capable of inducing Treg *in vitro*. By electroporation, PS antigen was induced on DC generated with our fast 3 day protocol and no severe cell death was observed. It was also reported in our former research that the 3-day DC are more robust to electroporation than traditional 7-day DC^30^.

Antigen-specific Treg were obtained in cultures using PS-antigen-loaded tolDC. In order to demonstrate the type of PSTreg, we use nTreg-depleted CD4^+^ T cells as precursor to generate PSTreg. As shown in the result, PSTreg can be generated with these cells, which indicated that in our method PSTreg are induced Treg. As expected, Foxp3 was highly expressed in PSTreg but also in NTreg that were generated using non-antigen-loaded tolDC. The development of NTreg may have resulted from exposure of T cells to high levels of exogenous IL-2 and rapamycin^34^. Foxp3 directly blocks SATB1 and indirectly induces microRNA which bounds to the *SATB1* 3′ untranslated region, therefore low *SATB1* expression was found to be characteristic for Treg and their suppressive function^37^. In concordance, the NTreg in our studies showed inhibited *SATB1* expression. While PSTreg had slightly upregulated expression of *SATB1* compared with PSTeff, they also showed reduced expression of *SATB1* compared with NTeff. GARP is a marker of activated Treg^38^, and can be used to isolate Treg with high suppressive function^38, 39^, and GARP associates with Foxp3 expression^40^. More importantly, GARP is involved in TGF-β expression by forming the GARP-LAP complex on the Treg surface^41^. In the cells studied here, higher expression of *GARP* was found in PSTreg compared to NTreg, indicating that foreign-antigen stimulation may activate Treg more efficiently than self-antigens. Consistently, a more memory phenotype was observed in our PSTreg, which showed CD45RA^−^CCR7^low^ phenotype^42^. With IL-10 producing tolDC, induced Treg can be generated and exhibit a more activated phenotype that was also reported by others^43^. The high CCR4 expression again confirmed the high suppressive function of PSTreg, which demonstrated by others that the CCR4 expressing CD45RA^−^FOXP3^high^CD4^+^ Treg are terminally differentiated and most suppressive^44^. The stability of our PSTreg was demonstrated upon PS antigen loaded tolDC, by Foxp3 expression, an activated phenotype and specific suppressive function. Fast porcine-specific tolDC provide an effective tool to successfully generate PSTreg.

High expression of IL-10 and TGF-β1 and the lack of IL-12p70, confirmed the tolerogenic phenotype. In contrast, C5-DC expressed IL-12p70, but not IL-10, in accordance with their immunogenic phenotype. Although *EBI3* mRNA expression showed no significant difference between tolDC and C5-DC in most samples *EBI3* mRNA was downregulated in tolDC, which can be explained by a previous report demonstrating that IFN-γ could induce *EBI3* expression in DC^45^. *IL-27A* expression was upregulated more in C5-DC compared to tolDC, which was also consistent with the former report^45^. In general, C5-DC, as immunogenic antigen-presenting cells, produce IL-27 and IL-12p70, but not IL-35. Our tolDC downregulate IL-27, and do not express IL-12p70, as another confirmation of the tolerogenic character of these cells.

As expected, PSTreg secreted high level of IL-10 and TGF-β1 at protein and RNA levels. Based on the mRNA expression of *EBI3* and *IL-12A*, it is evident that PSTreg were more strongly activated after exposure to foreign antigens compared with NTreg that were activated by self-antigens. Although compared with PSTeff *EBI3* upregulation in PSTreg met no statistical significance, *IL-12A* was significantly upregulated, which confirmed observations of others that human activated Treg upregulated predominantly more *IL-12A* than *EBI3* ^24^. We speculate that our fully activated PSTreg produce IL-35. Because there is no direct method to measure IL-35 as a dimeric protein, we can only infer IL-35 production indirectly from their *EBI3* and *IL-12A* mRNA expression. The functionality of PSTreg was clearly demonstrated: two activation markers of different activation stages were significantly suppressed on PSTeff by exposure to PSTreg compared with NTreg generated with mock-loaded tolDC and nTreg. Likewise, proliferation of PSTeff was inhibited significantly in the presence of PSTreg and PSTreg still retained the specific suppression towards PSTreg in lower ratio, indicating it is applicable *in vivo*. In contrast, PSTreg inhibited NTeff proliferation and the expression of activation markers of NTeff were significantly lower than in PSTreg compared to PSTeff, and in the ratio of 1:1, PSTreg showed no significant suppressive function to NTeff. This demonstrates that PSTreg generated with our method exhibit high suppressive activity only towards PSTeff and indicates that PSTreg are highly specific and do not mediate non-specific, unwanted immunosuppression. In comparison with nTreg isolated from the same donor at the same time, NTreg suppressive activity towards PSTeff and NTeff showed no significant difference to nTreg in the aspect of activation marker expression and Teff proliferation. NTreg showed higher suppressive activity than nTreg in the PSTeff CD25 expression assay. This indicates that NTreg generated with our mock-loaded tolDC exhibit comparable function to nTreg in the aspect of Teff suppression. Moreover, IL-17A production was not detected, and PSTreg still showed the specific suppressive function after restimulation, which indicates high stability of our PSTreg.

**Figure 6:**
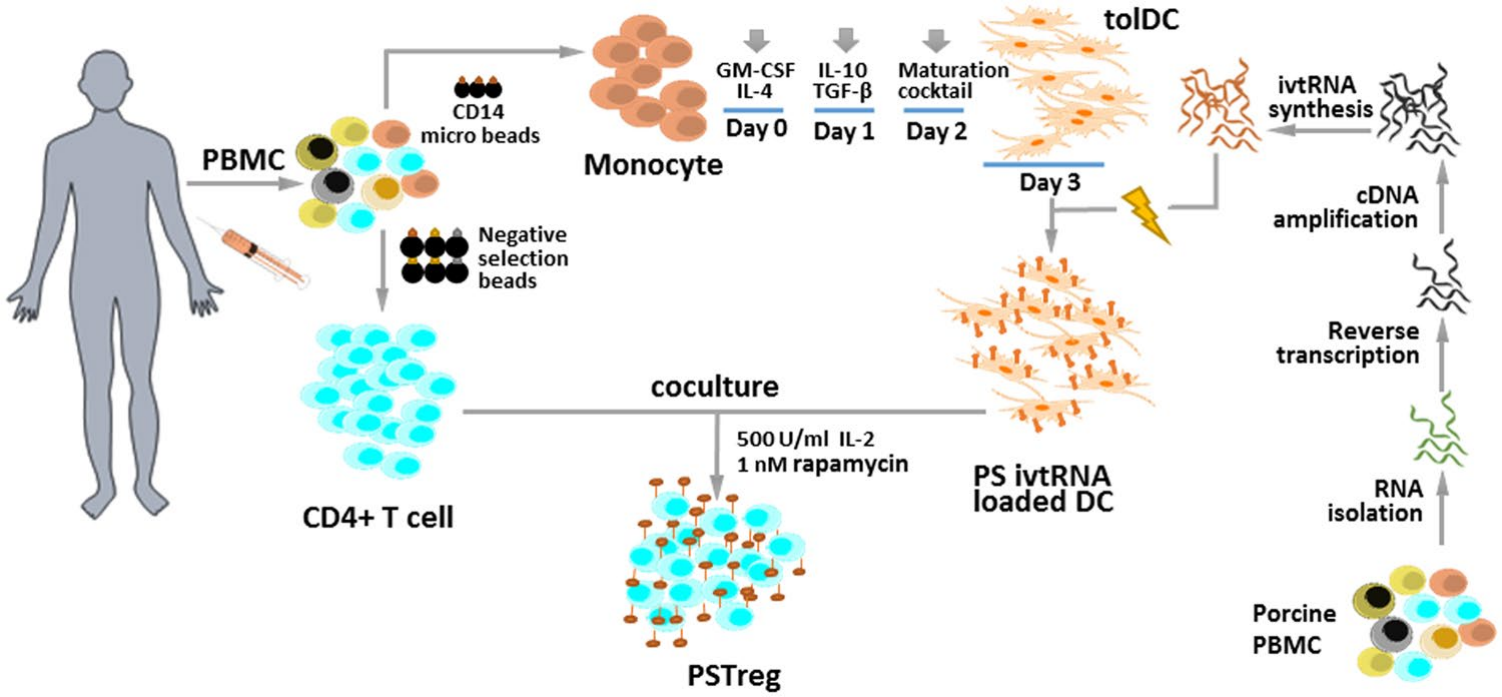
Flowchart of PSTreg generation.

IL-10 secretion was reproducibly observed during the first few hours of coculture of PSTreg and PSTeff, and became more pronounced after longer incubation periods. TGF-β1 also accumulated preferentially in PP cocultures, although differences with other coculture combinations were less prominent (Fig. 6). In line with protein levels, PP cocultures contained the highest amounts of *IL-10* and *TGFB1* mRNA. Interestingly, from the statistical aspect, the increase of *IL-10* mRNA in PP was more pronounced than that of *TGFB1* mRNA, which indicates that IL-10 might be the major cytokine of the two involved in PSTreg suppressive function. An additional potential candidate involved in suppressive function of PSTreg is IL-35, confirmed by earlier studies^21^. The mRNAs of IL-35 components *EBI3* and *IL-12A* were preferentially expressed in PSTreg upon interaction with PSTeff.

IFN-γ is a cytokine that mediates inflammation and causes potent immune regulatory effects. As expected, IFN-γ was highly expressed by PSTeff in the absence of PSTreg. Coculture of PSTeff with PSTreg strongly suppressed IFN-γ secretion. However, after extended coculture with PSTreg, IFN-γ production increased. Due to their plasticity Treg produce IFN-γ when they are recruited to the site of Th1-type inflammation, and there is evidence that IFN-γ also has immune inhibitory effects: it induces PD-L1^35^ and IDO^46^ expression in DC and upregulates the expression of *EBI3* ^45^. In addition, IFN-γ mediated protection in GvHD and closely associated with Treg development and function in GvHD sittings^47^. Therefore, it is conceivable that PSTreg profit from an IFN-γ environment produced by PSTeff through upregulation of *EBI3* expression, as shown in the results section.

Generally, Treg may inhibit cellular rejection in several ways: (a) by secretion of suppressor cytokines, such as IL-10 and TGF-β, which inhibit effector T cells directly, (b) by expression of high levels of CD25, leading to competition for IL-2 with effector T cells, (c) by acting as cytotoxic cells that directly kill responder T cells, and (d) by inducing expression of galectin-1 or other unknown molecules on the cell surface leading to effector T cell cycle arrest^48^. Thus, it is unlikely that PSTreg mediate their specific suppressive functions solely via secretion of anti-inflammatory cytokines. Further investigations will be required to elucidate the exact mechanisms that contribute to the distinct specificity of PSTreg described in these studies. Such analyses become feasible through the capacity of tolDC to induce these cells in a rapid and efficient manner, leading to generation of PSTreg with high stability.

PSTreg developed with this fast method represent Treg that exhibit a phenotype of activated cells and produce high levels of IL-10-, TGF-β1- and IL-35 and also have porcine-antigen specificity. In contrast to the methods used by others our method uses ivtRNA to induce xeno-antigen specific tolDC to generate PSTreg, which is safe and ivtRNA is easy to generate in large amounts. A recent study reports large-scale expansion of Treg with CD3/CD28 beads together with rapamycin and IL-2 and then the cells were restimulated additionally with irradiated pig PBMC, but the specificity was lost following several restimulations^49^. Our PSTreg generated with tolDC might provide a better protocol, but this should be demonstrated in the future. In the baboon system, enriched and expanded Treg can suppress xenogeneic immune responses, and it can be suggested that adoptive transfer of baboon Treg cells may be an approach to prevent xeno-graft rejection in a pig-to-baboon xenotransplantation model^50, 51^. Therefore, we successfully transferred our technique into the baboon system (unpublished results). This enables our method of tolDC generation with subsequent induction of porcine-specific Treg to be developed for use in porcine solid organ transplantation or porcine cell transplantation through adoptive cell transfer into host animals or human transplant patients in the future.

## Methods

### Human and porcine blood samples

Healthy human donors gave written informed consent before the experiments were done, approved by the Ethics Committee of the Ludwig Maximilians University Munich. Human serum samples were produced from blood of healthy male donors, even approved by the Ethics Committee. Porcine PBMC were from blood of wild type pigs, approved by the local authorities and the Government of Upper Bavaria. The animals received treatment in compliance with the Guide for the Care and Use of Laboratory Animals, published by the US National Institutes of Health (2011) and National Law. All methods and experiments were carried out in accordance with relevant guidelines and regulations, approved by the Committee of the LIFE Center of the Ludwig Maximilians University.

### RNA isolation and ivtRNA generation

RNA of porcine PBMC was isolated using the RNeasy Mini Kit (Qiagen, Germany) according to the manufacturer’s instructions. RNA quality and quantity was controlled by Agilent capillary electrophoresis (Agilent Technologies, USA) and Nanodrop (Thermo Scientific, USA), respectively. Reverse transcription of porcine PBMC RNA was accomplished with SMARTer™ PCR cDNA Synthesis Kit (Clontech Laboratories, USA). cDNA was amplified by Advantage® 2 PCR Enzyme System (Clontech Laboratories, USA). The following primers were used: 5′ primer/T7: 5′-TAATACGACTCACTATAGGGAGGAAGCAGTGGTAACAACGCA-3′ 3′ CDS primer: 5′-AAGCAGTGGTATCAACGCAGAGT-3′. ivtRNA of porcine PBMC was generated by mMESSAGE mMachine T7 Ultra Kit (Life Technologies, USA) and purified by MEGAclear™ Transcription Clean-Up Kit (Life Technologies, USA). The full length capped mRNA was analyzed and quantified using the Agilent system (see also Fig. S2 supporting information).

### TolDC and mature C5-DC generation

Monocytes were isolated from PBMC of healthy donors using CD14 microbeads (Miltenyi Biotec, Germany). The cells were resuspended in VLE-RPMI 1640 (Biochrom AG, Germany) with 1.5% human serum and seeded at 5 × 10^6^ cells in 25 ml Nunclon™flasks (Nunc, Germany). On day 0, 100 ng/ml GM-CSF and 20 ng/ml IL-4 were added to the cultures. At day 1, 1 ng/ml TGF-β1 and 20 ng/ml IL-10 were added only to tolDC. On the following day, maturation cocktails^30^ (Table 2) were added to tolDC and C5-DC to induce maturation. For immunophenotyping of tolDC and C5-DC, the following antibodies were used: anti-CD14 (clone: M5E2), anti-CD80 (clone: L307.4), anti-CD83 (clone: HB15e), anti-B7-H1 (clone: MIH1), anti-B7-DC (clone: MIH18) (all from BD Biosciences, USA) and anti-CD86 (clone: 2331, Pharmingen). Stained cells were analyzed by using the LSRII (BD Biosciences). Data were processed by using FlowJo software (Tree Star, Ashland OR, USA).

**Table 2:** Maturation cocktail for generation of tolDC and C5-DC.

	TolDC	C5-DC
GM-CSF (Leukine sargramostim, USA)	100 ng/ml	100 ng/ml
IL-4 (R&D, USA)	20 ng/ml	20 ng/ml
IL-1β (R&D, USA)	10 ng/ml	10 ng/ml
TNF-α (PEPRO-TECH, USA)	20 ng/ml	20 ng/ml
PGE_2_ (Sigma, USA)	250 ng/ml	1 µg/ml
R848 (*In vivo* Gen)	—	1 µg/ml
IFN-γ (Boehringer Ingelheim, Germany)	—	5000 U/ml
IL-6 (R&D, USA)	15 ng/ml	—
TGF-β1 (PEPRO-TECH, USA)	1 ng/ml	—
IL-10 (PEPRO-TECH, USA)	20 ng/ml	—

### Loading of DC with porcine ivtRNA

Electroporation of DC was done in 0.4 cm electroporation cuvettes using the Gene Pulser Xcell™ (Bio-Rad Laboratories GmbH, Germany). 1.5 × 10^6^ DC were resuspended in 200 µl Opti-MEM (Gibco, Life Technologies, USA) and incubated with 10 µg ivtRNA for 5 minutes on ice. For electroporation the following conditions were used: exponential protocol, 150 µF, 300 V. DC electroporated in the presence of PBS served as controls. Immediately after electroporation, the cuvettes were placed on ice for 5 minutes and then the DC were cultured with VLE-RPMI 1640 plus 1.5% human serum at 37 °C and 5% CO_2_ for 24 h. Expression of porcine PBMC ivtRNA was assessed by detection of porcine MHC-class I protein (porcine MHC-class I antibody, gift from R. Kammerer).

### Generation PSTreg and PSTeff

Human CD4^+^ cells were isolated using the CD4^+^ T Cell Isolation Kit (Miltenyi Biotec, Germany), cocultured with porcine RNA-loaded human tolDC at the ratio of 1:10. To isolate or deplete nTreg, CD4^+^CD25^+^ Regulatory T Cell Isolation Kit (Miltenyi Biotec, Germany) was used. Human PSTeff were generated from cocultures of porcine antigen-loaded C5-DC^44^ and CD4^+^ T cells. NTreg and NTeff cells were generated using mock-loaded tolDC and C5-DC, respectively. The T cells were incubated for 10 days supplemented with IL-2 (Treg: 500 U/ml Teff: 50 U/ml) and with or without rapamycin (Treg: 1 nM). To restimulate the cells were harvested, and PSTreg and NTreg were purified with CD4^+^CD25^+^ Regulatory T Cell Isolation Kit (Miltenyi Biotec, Germany), and cocultured with DC as mentioned above for 10 days. The Treg phenotype was characterized by flow cytometry (LSRII, BD Biosciences). The following monoclonal antibodies were used: anti-CD3 (clone: SP34-2, BD), anti-CD4 (clone: L200, BD Biosciences), anti-CD25 (clone: BC96, eBioscience, USA), anti-CD127 (clone: MB15-18C9, Miltenyi Biotech, Germany), anti-Foxp3 (clone: PCH101, eBioscience), anti-CD45RA (clone:5H9, BD), anti-CCR7 (clone: G043H7, Biolegend) anti-CCR4 (clone: 1G1, Pharmingen). Intracellular staining for Foxp3 was performed by Foxp3 staining buffer set (eBioscience). LIVE/DEAD® Fixable Blue Dead Cell Stain Kit (Life Technologies, USA) was used to determine viable cells and to exclude dead cells. The procedure for generation of PSTreg is summarized in the flowchart depicted in Fig. 6.

### PSTreg functional assays

Three assays were used to test the suppressive function of PSTreg: inhibition of Teff early activation marker (CD154) expression^52^, inhibition of Teff proliferation, and inhibition of CD25 expression.

Treg cells and Teff cells were stained separately with the Vybrant® DiI Cell-Labeling Solution (Life Technologies) and with CFSE (CellTrace™ CFSE Cell Proliferation Kit, LifeTechnologies), according to the manufacturer’s instructions. 1 × 10^5^ PSTeff and PSTreg were seeded at different ratios in 96 U-bottom wells (PP). CD3/28 beads were then added at the ratio of 1:4 according to cell numbers. PSTeff + NTreg (PN), NTeff + PSTreg (NP), NTeff + NTreg (NN), PSTeff + nTreg (PSTeff-nTreg) and NTeff + nTreg (NTeff-nTreg) were set up as Treg and Teff controls, PSTeff (P) and NTeff (N) as Teff controls.

After 7 h and 96 h incubation, cells were harvested and stained for CD154 (clone: TRAP1, BD) and CD25 (clone: M-A 251, Pharmingen) respectively. Teff proliferation was tested after 96 h. In all assays, LIVE/DEAD® Fixable Blue Dead Cell Stain Kit (Life Technologies) was used to determine the viability and exclude the dead cells during analysis.

### Cytokine production

Supernatants of DC, PS/NTreg and PS/NTeff cultures, and PP, PN, NP, NN cocultures were harvested to determine cytokine production. Secreted IL-12p70, IL-10 and TGF-β1 levels were measured with ELISA (R&D, USA) and BD™ CBA Flex Set system (BD Biosciences) according to the manufacturers’ instructions. IL-17a and IFN-γ were quantified with BD™ CBA Flex Set system (BD Biosciences) according to the manufacturer’s instructions.

### Reverse transcription polymerase chain reaction (RT-PCR)

Tol/C5-DC, PS/N Treg/Teff and PP, PN, NP, NN were harvested and washed twice with PBS. RNA was isolated as described above. Reverse transcription was accomplished using the Reverse Transcription System (Promega, USA), according to the manufacturer’s instructions.

Primers for quantification of *Foxp3*, *STAB1*, *GARP*, *EBI3*, *IL-12A*, *IL-27A* cDNA are compiled in Supporting Information Table S1. Primers for quantification of *IL-12B*, *IL-10*, *TGFB1* and housekeeping genes *ACTB*, *G6PDH* and *cyclophilin B* (*CYPB*) cDNA were purchased from Search LC (Germany). RT-PCR was performed with FastStart Essential DNA Green Master (Roche, Germany) and Light Cycler 96 (Roche). The quality of the PCR primers was confirmed by melting curve analysis (Supporting Information Fig. S9).

The relative amounts of the cDNA content of interest in the unknown samples were calculated with the Livak method: relative expression of cDNA of interest was normalized with the control sample, and target gene relative expression was normalized by the reference gene.

$$relative\,normalized\,expression\,ratio={2}^{(-(\begin{array}{c}(Cq(target,unknow)-Cq(reference,unknow))\\ -(Cq(target,control)-Cq(reference,control))\end{array}))}$$

Cq represents the crossing point where a fluorescence value of one is reached.

### Statistical analysis

Data are displayed as mean ± SEM (standard error of the mean). Significance of data was analyzed by ONE-Way Analysis Of Variance with Bonferroni’s Multiple Comparison Test, or turkey test, or Wilcoxon signed-rank test in Graphpad Prism 5.00 software (San Diego, USA).

## Electronic supplementary material

Supplementary Information
