# Supplementary material for: A novel and effective method to generate human porcine-specific regulatory T cells with high expression of IL-10, TGF-β1 and IL-35
Source: Sci Rep. 2017 Jun 21;7:3974. doi: 10.1038/s41598-017-04322-3 (PMC5479824; doi:10.1038/s41598-017-04322-3)
Supplement: Supplementary file 1 — Supplementary Information [file 41598_2017_4322_MOESM1_ESM.pdf]

Supporting information

A novel and effective method to generate human porcine-specific regulatory T cells with high expression of IL-10, TGF-β1 and IL-35

Mingqian Li, Judith Eckl, Christiane Geiger, Dolores J. Schendel, Heike Pohla

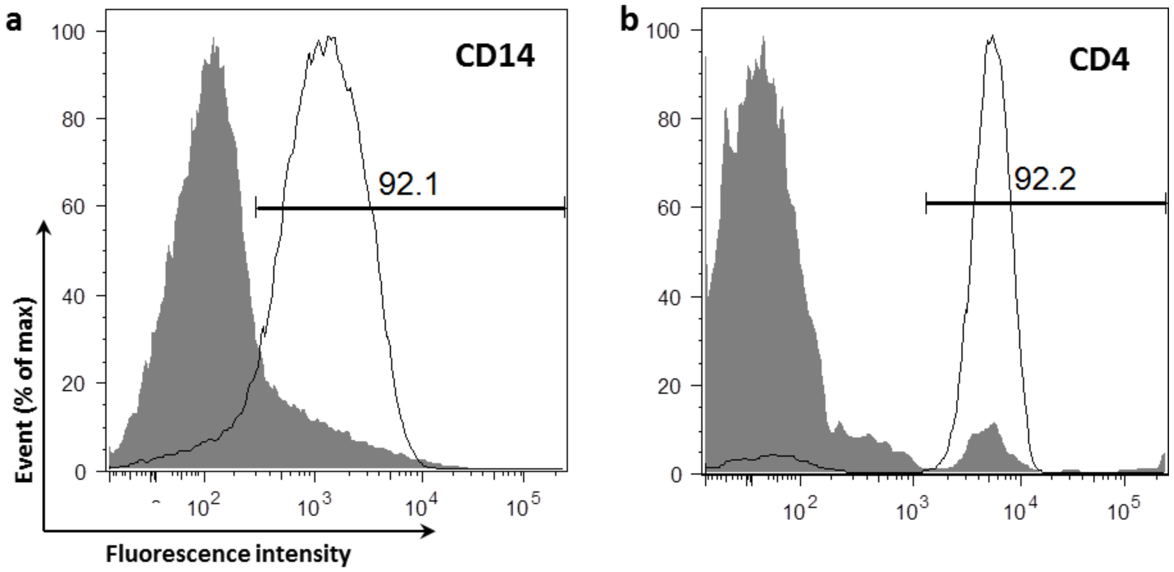

Supplementary Figure S1: Isolation of monocytes and CD4$^+$ cells from human PBMC. (a) Monocytes were isolated from human PBMC using CD14 microbeads by positive selection. The purity of CD14$^+$ cell was analyzed by flow cytometry. (b) CD4$^+$ cells were isolated using the CD4 isolation kit by negative selection. The purity of CD4$^+$ cell was tested by flow cytometry.

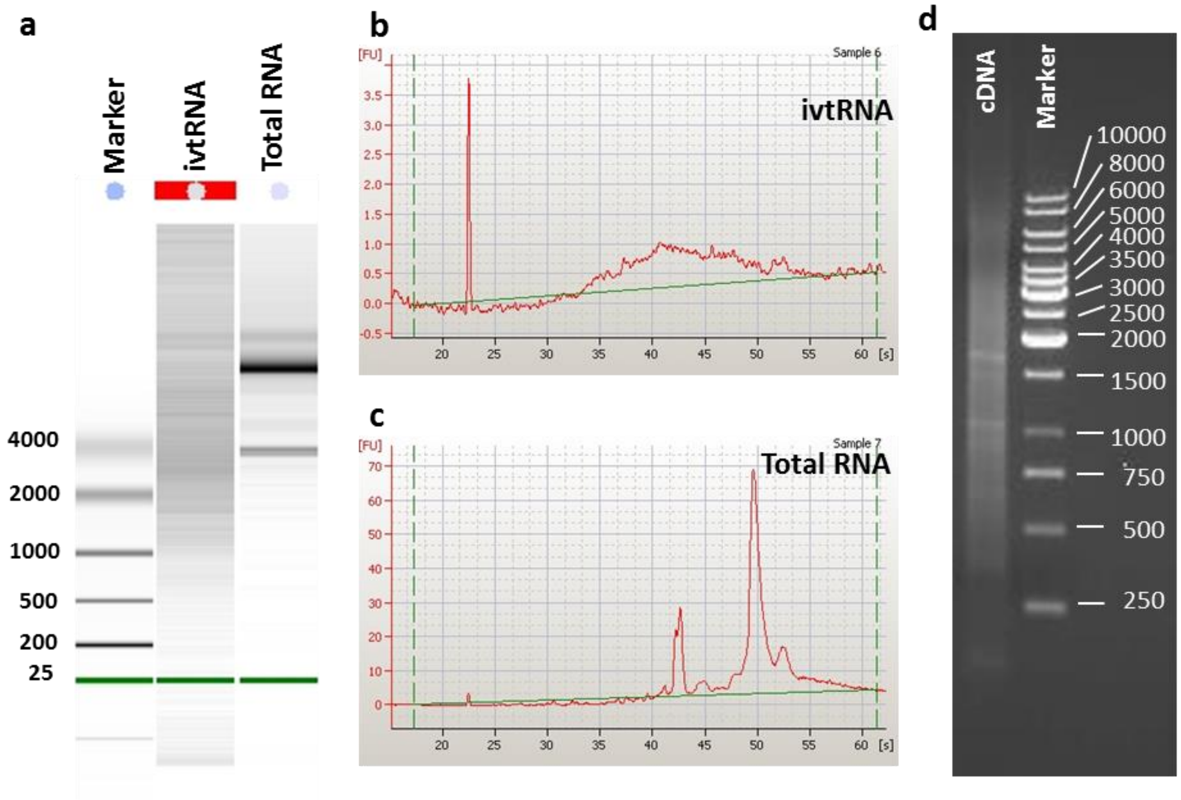

Supplementary Figure S2: Generation of porcine ivtRNA. Porcine PBMC RNA was isolated using the RNeasy Mini Kit, cDNA was synthesized using reverse transcription with SMARTer™ PCR cDNA Synthesis Kit and the Advantage® 2 PCR Enzyme System. The following primers were used: 5' primer/T7: 5'-TAATACGACTCACTATAGGGAGGAAGCAGTGGTAACAACGCA-3' 3' CDS primer: 5'-AAGCAGTGGTATCAACGCAGAGT-3'. Then ivtRNA was generated by mMESSAGE mMachine T7 Ultra Kit. The quality of porcine PBMC RNA (a, c) and ivtRNA (a, b) was tested by Agilent. The cDNA (d) quality was tested by agarose gel electrophoresis.

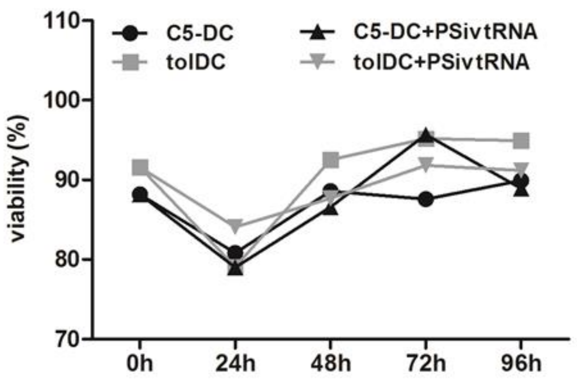

Supplementary Figure S3: Viability of TolDC and C5-DC after electroporation of porcine-specific ivtRNA.

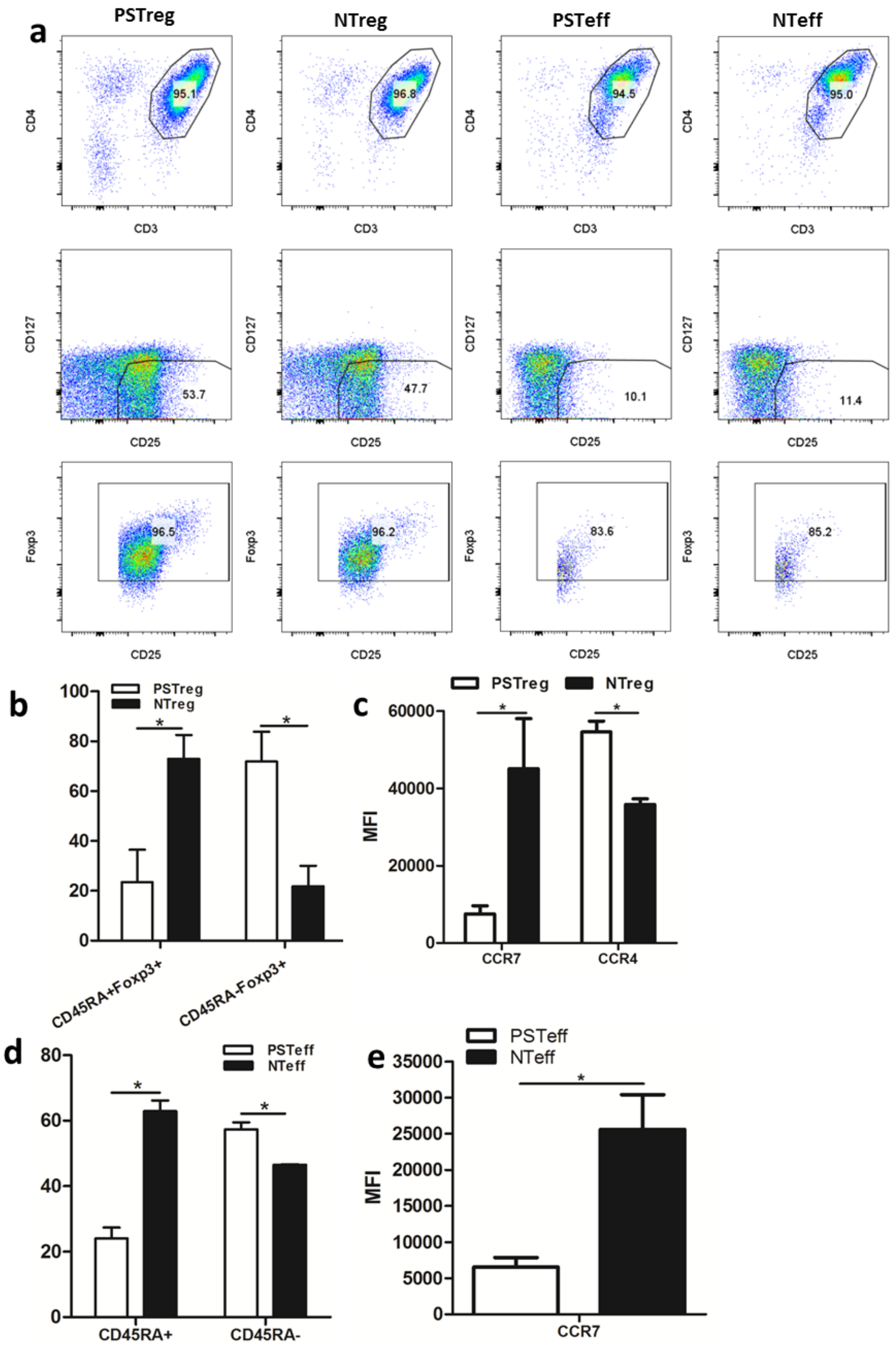

Supplementary Figure S4: PSTreg and PSTeff can be generated with porcine antigen loaded DC. (a) shows Treg gating strategy, PSTeff and NTeff are shown as control. (b) Percentage of CD45RA$^+$Foxp3$^+$ and CD45RA$^-$Foxp3$^+$ populations in PSTreg and NTreg. (c) CCR7 and CCR4 MFI of PSTreg and NTreg. (d) Percentage of CD45RA$^+$ and CD45RA$^-$ in PSTeff and NTeff. (e) CCR7 MFI of PSTeff and NTeff. n=3.

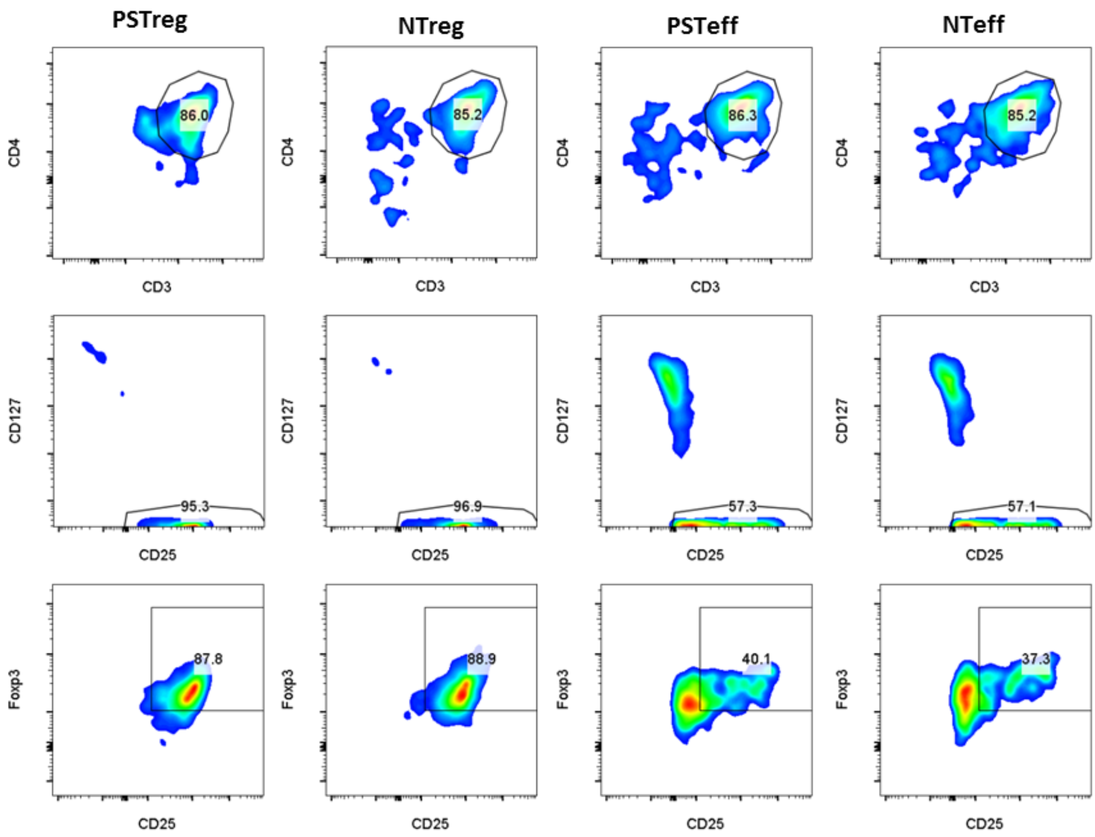

Supplementary Figure S5. Gating strategy for PSTreg and NTreg after restimulation for 10 day. PSTeff and NTeff are shown as controls.

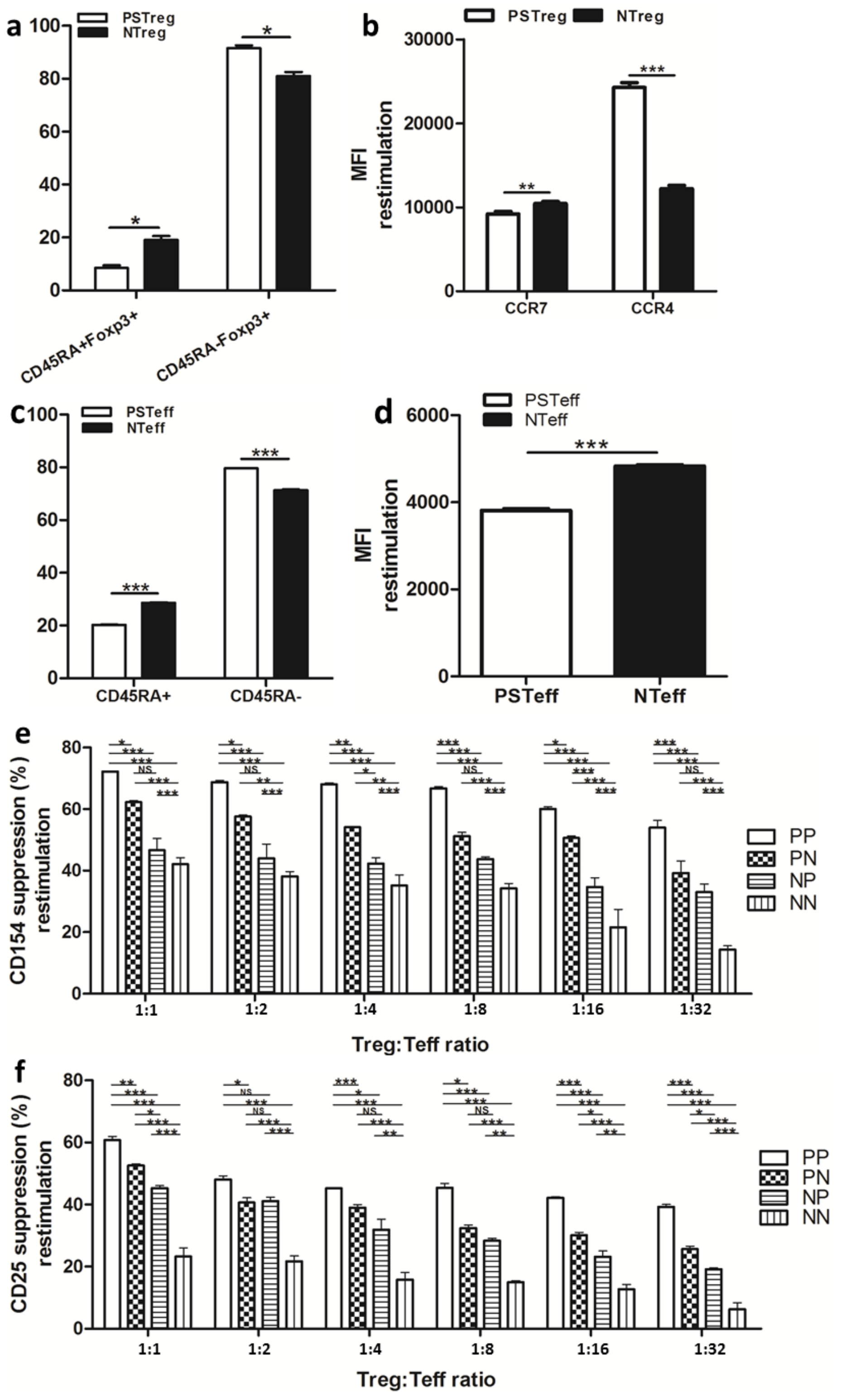

Supplementary Figure S6. PSTreg retained Foxp3 expression and specificity after restimulation. PSTreg, NTreg and PSTeff, NTeff were harvested after cocultured with DC for 10 days. Then PSTreg and NTreg were purified, and all cells were restimulated again with DC for 10 days. (a) Percentage of $CD45RA^{+}Foxp3^{+}$ and $CD45RA^{-}Foxp3^{+}$ populations in PSTreg and NTreg after restimulation. (b) CCR7 and CCR4 MFI of PSTreg and NTreg after restimulation. (c) Percentage of $CD45RA^{+}$ and $CD45RA^{-}$ in PSTeff and NTeff after restimulation. (d) CCR7 MFI of PSTeff and NTeff after restimulation. The specificity was tested by suppression of Teff activation marker at different ratios after restimulation, (e) CD154 suppression ratio, (f) CD25 suppression ratio. n=3.

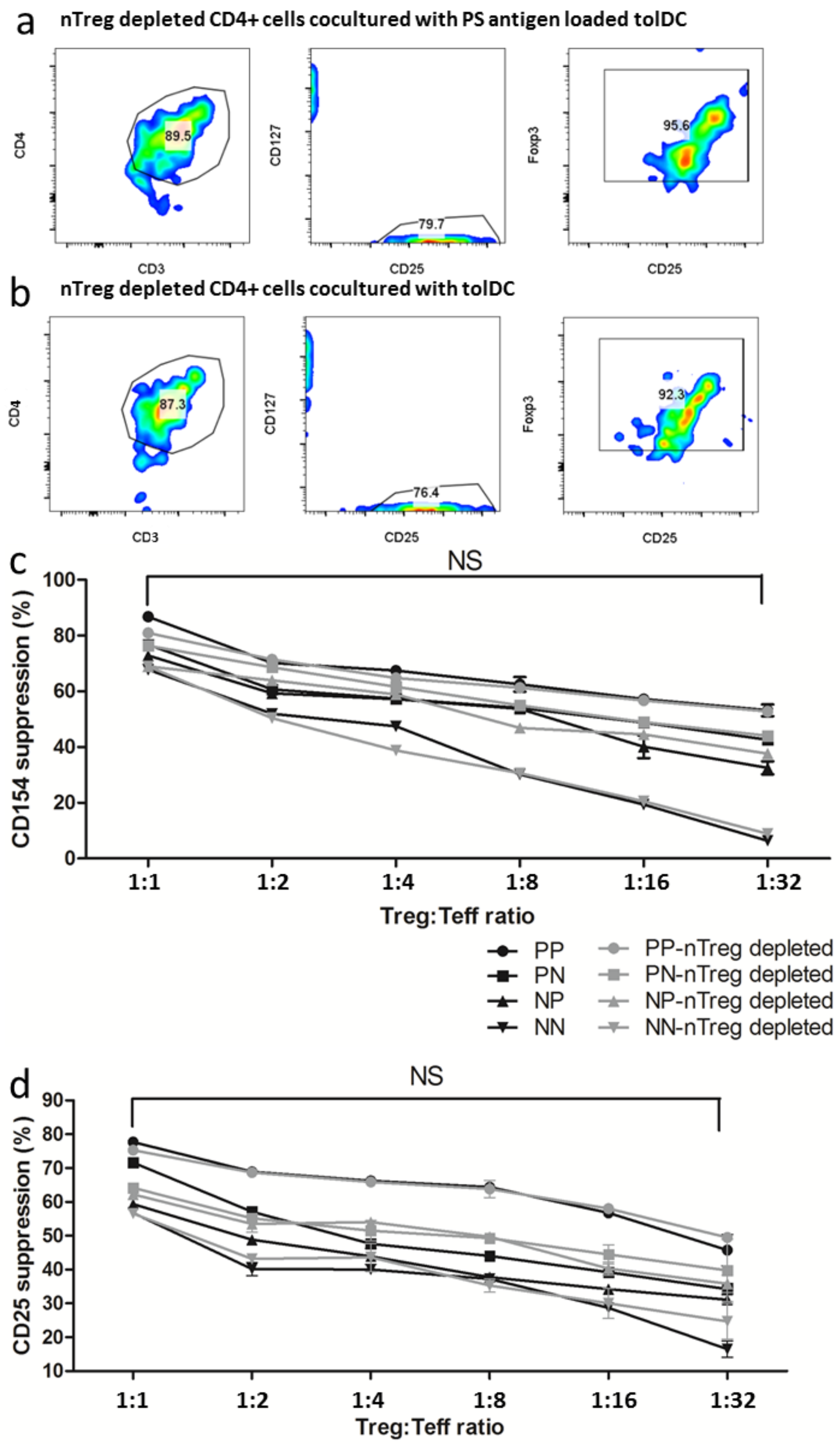

Supplementary Figure S7: Generation of PSTreg using nTreg depleted CD4^{+} cells as precursors. After depletion of nTreg, residual CD4^{+} cells were cocultured with PS antigen loaded or mock loaded tolDC or C5-DC. After coculture for 10 days, the cells were harvested and phenotyped. (a, b) show the gating strategy of PSTreg and NTreg generated from nTreg depleted CD4^{+} cells. Suppression assays were set up and the comparison of nTreg depleted and non-depleted CD4^{+} cell induced PSTreg function is shown in (c, d).

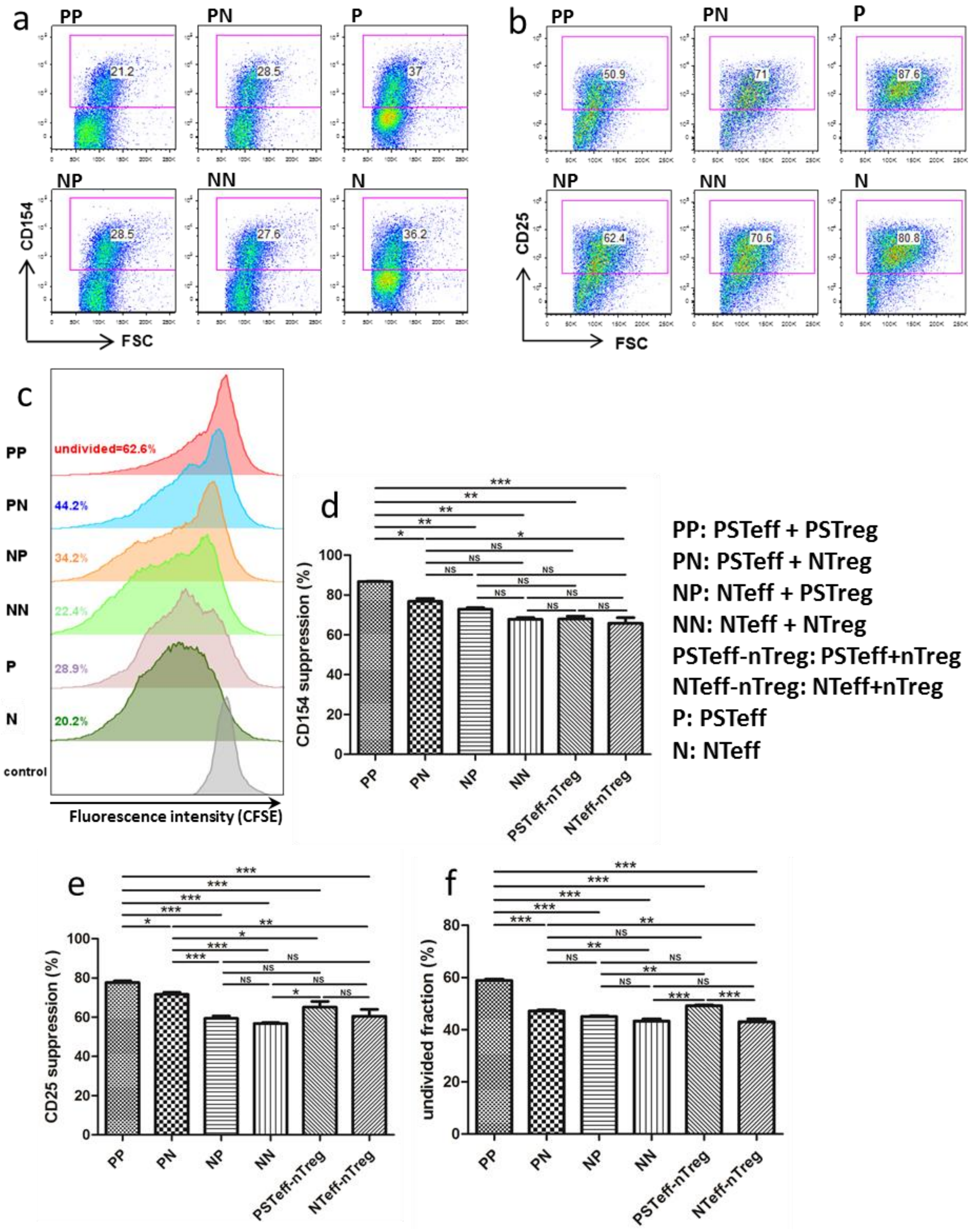

Supplementary Figure S8: PSTreg show specific immunosuppressive activity. Three different assays of PS/NTreg and PS/NTeff at a ratio of 1:1 are shown, (a) CD154 suppression, (b) CD25 suppression, (c) suppression of Teff proliferation. nTreg were used as control for PSTreg specificity: (d) CD154 suppression, (e) CD25 suppression, (f) shows Teff undivided fraction in the Teff proliferation assay. ONE-Way Analysis Of Variance with Bonferroni's Multiple Comparison Test was used to determine the statistical significance. n=3

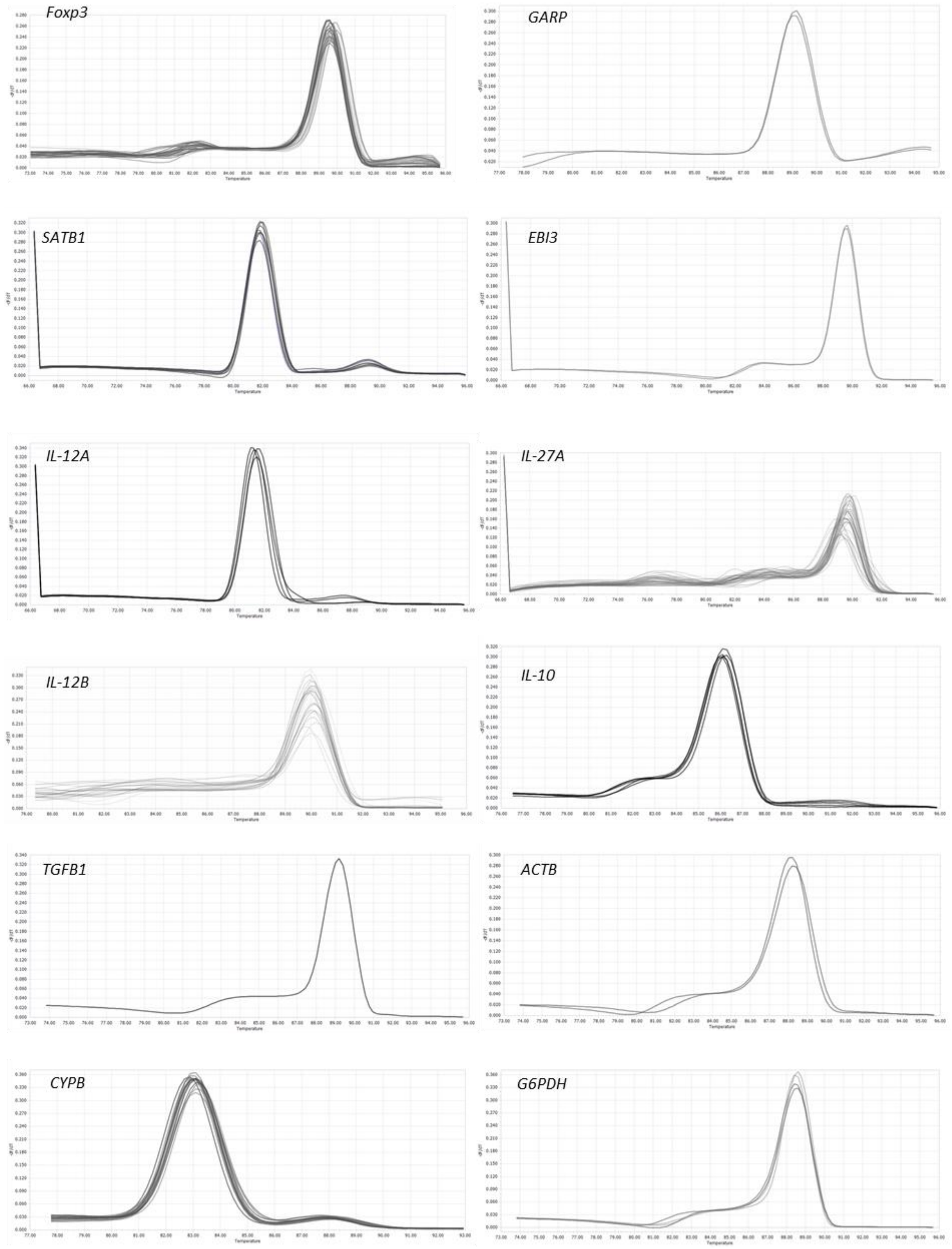

Supplementary Figure S9: Melting curves of RT-PCR to confirm the RT-PCR quality.

Supplementary Table S1. Sequences of primers used for RT-PCR

	Forward primer	Reverse primer
Foxp3	AGCCATGATCAGCCTCACAC	GACACCATTTGCCAGCAGTG
SATB1	CACTCGGGCCATCTGATGAA	GGGCAGCAGAGCTATGTGAAT
GARP	GCTTGACCTGCATAGCAACG	CCGGATGAGGTTGTTGGACA
EBI3	GCTCCCTACGTGCTCAATGT	CCCTGACGCTTGTAACGGAT
IL-12A	TGGCCCTGTGCCTTAGTAGT	GTTTGGAGGGACCTCGCTTT
IL-27A	GCCAGGAGTGAACCTGTACC	CACAGCTGCATCCTCTCCAT
